# The Myanmar military coup: Propelling the 2030 milestones for neglected tropical diseases further out of reach

**DOI:** 10.1371/journal.pntd.0009532

**Published:** 2021-07-15

**Authors:** Bethany Moos, Russell Roberts, Mo Aye

**Affiliations:** 1 Hedena Health, Oxford, United Kingdom; 2 Royal College of Physicians and Bradford Teaching Hospitals NHS Foundation Trust, Bradford, United Kingdom; 3 Hull University Teaching Hospitals NHS Trust, Hull, United Kingdom; Swiss Tropical and Public Health Institute, SWITZERLAND

On February 18, 2021, the world witnessed the touchdown of National Aeronautics and Space Administration (NASA’s) Perseverance rover on Mars. The people of Myanmar took pride that a Burmese-American engineer led the NASA’s Ingenuity project that flew a helicopter in thin Martian air 300 million miles away [1]. Alongside this space exploration, are we simultaneously watching Myanmar disappear into a black hole, devoid of light and hope? The Tatmadaw (Myanmar’s military), led by General Min Aung Hlaing, staged a coup on February 1, 2021, putting an end to a brief experiment with democracy. Hotez highlights political instability and collapse as one of the Anthropocene forces that threatens the significant gains made on neglected tropical diseases (NTDs) [2]. There are so many examples of how political instability drives poverty, undermines a health system, and contributes to poor health outcomes; yet, sadly, there is no need to look beyond Myanmar itself. The impact of the previous military regime is clear for all to see, with the incidence of catastrophic healthcare expenditure ranging from 6% to 18% in 2015 [3]. Now, we face the reality of history repeating itself. NTDs have been so grouped due to the fact that they disproportionately affect rural impoverished communities. The term “neglect” derives from the Latin verb “neglegere,” literally meaning “to disregard” or “to ignore.” The blatant disregard for human rights by the military junta threatens to push already marginalized communities, and, indeed, the whole country, into an abyss. We must not ignore this issue, and we cannot omit to consider the ramifications for generations to come.

Myanmar is a rural agricultural country in Southeast Asia where many NTDs prevail; notably, snakebite envenoming and rabies remain endemic, contributing to a considerable burden of morbidity and mortality. Additionally, dengue and soil-transmitted helminthiases remain prevalent. It remains commonplace to seek help from a traditional healer as many barriers to accessing healthcare in Myanmar prevail. A review of progress toward universal health coverage in Myanmar highlighted “low coverage of health services, high financial risk because of out-of-pocket payments, and large inequalities” as obstacles to achieving this goal by 2030 [3]. Furthermore, health-seeking behaviors are deeply rooted in traditional health beliefs, which vary between the different ethnic groups, of which there are over 100. Tourniquet use, cutting, and tattooing around a bite site are frequently used to treat snakebite envenoming [4]. Not only do such treatments delay access to appropriate medical care, but they are also detrimental in their own right. Community engagement is key to sustainable behavior change; however, it is not sufficient in isolation to improve outcomes in Myanmar. Adequate infrastructure, staffing and funding are integral components of a healthcare system that is not only functional, but is also thriving. The military coup compromises all of these. Our medical colleagues have been directly targeted by the military junta, and vital lifesaving equipment has been destroyed or removed from hospitals.

“Ending the neglect to attain the Sustainable Development Goals: a road map for neglected tropical diseases 2021–2030” outlines the WHO targets to be achieved by 2030 [5]. It calls for expedited programmatic action with an emphasis on cross-sectoral collaboration and for countries to take the lead in the control, eradication, and elimination of NTDs. Urgent action is required to combat rabies in both humans and animals in Myanmar as it is now more common than malaria [6]; the WHO target of achieving zero human deaths secondary to dog-mediated rabies in Myanmar is certainly in jeopardy [7]. Recent impressive gains in other NTDs such as lymphatic filariasis, soil-transmitted helminthiases, and leprosy are now in serious jeopardy. Many have taken refuge at the Thai–Myanmar border; this is of concern given that this is the region where falciparum malaria with artemisinin resistance was first reported, and there remains a high prevalence of bancroftian filariasis [8,9]. The incidence of snakebite envenoming in Myanmar is 116 per 100,000, predominantly secondary to Russell’s viper, which is also notorious for causing a significant burden of acute kidney injury [10]. WHO aspires to halve death and disability from snakebite envenoming by 2030 through four strategic aims: through empowering and engaging communities, ensuring access to safe and effective treatments, strengthening health systems, and increasing partnerships, coordination, and resources [11]. The military coup threatens each of these four fundamental pillars and we use these constructs to demonstrate the potential impact not only on snakebite envenoming, but also on achieving the wider NTD targets in Myanmar.

## Community engagement and empowerment

Democracy signifies “the rule of the people”; the coup d’état has removed the voice of the people of Myanmar and is the strongest form of disempowerment. Instilling a regime of fear will affect the ability to engage with communities to improve health outcomes. Given the military now have an intimidating presence in many hospitals throughout the country, this is likely to have a significant impact on health-seeking behavior. There have been reports of the medical community being targeted, and at times prevented, from providing care. At least 85 doctors have been arrested since the coup began [12]. Already under siege from the efforts of responding to a global pandemic, the psychological and emotional toll on healthcare professionals could have a catastrophic impact on their ability to prop up a healthcare system under severe strain.

## Ensuring access to safe and effective treatments

Myanmar is fortunate to have locally produced lyophilized snake antivenom for cobra and Russell’s viper; however, it may well struggle to access other vital medicines for the control of NTDs, such as medications for mass drug administration and time-critical rabies post-exposure prophylaxis. This may drive individuals, particularly in remote and rural areas, to preferentially frequent a traditional healer over visiting a healthcare facility. Consideration should also be paid to the impact on access to appropriate medical treatments for both companion animals and livestock. This is essential not only because of the direct impact on human health, but also because the loss of livestock can be financially crippling for a rural farming community. It should also be noted that the response to the severe acute respiratory syndrome coronavirus 2 (COVID-19) pandemic, including the implementation of the vaccination program, has been severely disrupted secondary to the coup [13].

## Strengthening health systems

The Myanmar healthcare system has been systematically undermined by the previous military leadership, which leaves it at risk of “profound health system collapse” [13]. The coup has compounded the effect of a weakened healthcare system secondary to the previous regime and the toll of the COVID-19 response. Sadly, as the security forces respond to peaceful protests with lethal force, the relatively novel specialty of Emergency Medicine now has to face an increasing burden of trauma. Those in primary care have also had to step up and respond to trauma, far from their scope of usual practice. Meanwhile, doctors and nurses have joined the Civilian Disobedience Movement, saying that they “refuse to obey any orders from the illegitimate military regime who demonstrated they do not have any regard for our poor patients” [14]. Rather than accelerated action and collaboration, we are likely to see regression and division if this political instability continues. Given the political climate, the ability of the country to respond to demands, such as the prevention and control of COVID-19, future outbreaks of infectious diseases, or environmental disasters such as Cyclone Nargis, is likely to be severely limited. The latter illustrates just how ill-suited a repressive military regime is to the challenge of a major public health disaster.

## Increasing partnerships, coordination and resources

Myanmar has historically had many partnerships with external organizations, and the future of such alliances remain uncertain under this regime. The military junta casts doubt on securing and maintaining funding for existing and future partnerships. There is understandable concern from donor organizations, and, indeed, caution should be taken to ensure that funding is not inadvertently fueling the military regime. Given the precipice the country is balanced on, the importance of partnerships cannot be underestimated. The NTD Roadmap calls for in-country leadership; at this challenging time, collaboration to support the healthcare leaders in Myanmar will be critical in the fight against NTDs, specifically in the context of building back better from the COVID-19 pandemic and also to the continuation of basic medical services. Moreover, internet shutdowns and the suspicion with which the military views those in contact with the outside world severely compromises any international collaboration at the current time.

It is with great alarm that we observe the current political situation in Myanmar and the conditions that our colleagues are facing there. This should be of grave concern to the NTD community, as Myanmar faces not only a military coup, but also recovery from the COVID-19 pandemic. In our view, it will be impossible for any progress to be made in the battle against important NTDs in the current political situation, and we call for our international colleagues to support the return of democracy in Myanmar. The abuse of human rights and specific targeting of medical personnel and hospitals must be stopped immediately, and patients should be able to access appropriate healthcare without fear and intimidation.
